# The redox language in neurodegenerative diseases: oxidative post-translational modifications by hydrogen peroxide

**DOI:** 10.1038/s41419-020-03355-3

**Published:** 2021-01-11

**Authors:** Yew Mun Lee, Weifeng He, Yih-Cherng Liou

**Affiliations:** 1 Department of Biological Sciences, Faculty of Science, National University of Singapore, 14 Science Drive 4, Singapore, 117543 Singapore; 2 Institute of Burn Research, State Key Laboratory of Trauma, Burn and Combined Injury, Southwest Hospital, Army Medical University, No. 29 Gaotanyan Street, Shapingba District, Chongqing, 400038 China; 3 NUS Graduate School for Integrative Sciences and Engineering, National University of Singapore, Singapore, 117573 Singapore

**Keywords:** Post-translational modifications, Neurodegenerative diseases

## Abstract

Neurodegenerative diseases, a subset of age-driven diseases, have been known to exhibit increased oxidative stress. The resultant increase in reactive oxygen species (ROS) has long been viewed as a detrimental byproduct of many cellular processes. Despite this, therapeutic approaches using antioxidants were deemed unsuccessful in circumventing neurodegenerative diseases. In recent times, it is widely accepted that these toxic by-products could act as secondary messengers, such as hydrogen peroxide (H_2_O_2_), to drive important signaling pathways. Notably, mitochondria are considered one of the major producers of ROS, especially in the production of mitochondrial H_2_O_2_. As a secondary messenger, cellular H_2_O_2_ can initiate redox signaling through oxidative post-translational modifications (oxPTMs) on the thiol group of the amino acid cysteine. With the current consensus that cellular ROS could drive important biological signaling pathways through redox signaling, researchers have started to investigate the role of cellular ROS in the pathogenesis of neurodegenerative diseases. Moreover, mitochondrial dysfunction has been linked to various neurodegenerative diseases, and recent studies have started to focus on the implications of mitochondrial ROS from dysfunctional mitochondria on the dysregulation of redox signaling. Henceforth, in this review, we will focus our attention on the redox signaling of mitochondrial ROS, particularly on mitochondrial H_2_O_2_, and its potential implications with neurodegenerative diseases.

## Facts

- Mitochondria are one of the major producers of ROS in the cellular environment.
- Mitochondria possess an extensive antioxidant defense to maintain redox homeostasis.
- ROS, such as hydrogen peroxide, are increasingly viewed as important redox signaling molecules.
- Mitochondrial dysfunction is implicated in many neurodegenerative diseases.
- Increasing oxidative stress through aging is associated with many neurodegenerative diseases.

## Open questions

- Do the mitochondria produce ROS, such as hydrogen peroxide, to mediate cellular redox signaling?
- Is there a central role for mitochondria in the cellular redox signaling cascade?
- Does mitochondrial dysfunction disrupt the cellular redox signaling landscape?
- Is the disruption to the cellular redox signaling landscape that is mediated by mitochondrial dysfunction a trigger of neurodegenerative diseases, or is it a secondary effect from the manifestation of neurodegenerative diseases?

## Introduction

Growing evidence from various studies highlights the importance of mitochondria in disease manifestation, especially in aging and neurodegenerative diseases^1,2^. A long-standing viewpoint is the contribution of mitochondria to oxidative stress during aging: the decline of mitochondrial function leads to redox imbalance, which results in an increased production of reactive oxygen species (ROS) and the loss of cellular antioxidant defense; the subsequent overload of ROS induces mitochondrial dysfunction and triggers the apoptotic cascade. This scenario has been well documented in various neurodegenerative diseases^3^. Moreover, ROS dysregulation in microglia (resident brain macrophages that protect against brain damage) strongly correlates to increased oxidative stress and, consequently, neuronal death^4,5^. However, ROS do more than inducing cell death, they also contribute to cellular signaling.

ROS are chemical entities possessing a radical nature; they trigger chemical reactions that could lead to deleterious biochemical changes to the cellular environment^6^. Detailed documentation of the radical processes triggered by ROS has been well-reviewed elsewhere^7^. Recently, the effects of ROS have been shown to extend beyond their destructive nature^8^, as various species of ROS target cellular signaling by acting as important secondary messengers^9^. Notably, a review by Sies highlights superoxide (O_2_^•−^) and hydrogen peroxide (H_2_O_2_) as secondary messengers in various signaling pathways^10^, though H_2_O_2_ is considered more important owing to its higher stability and diffusive nature.

Evidently, ROS, particularly mitochondrial ROS (mtROS), contribute to neurodegenerative disease onset and progression *via* both redox imbalance and cellular signaling. Thus, this review will present a more detailed and up-to-date summary of mtROS-mediated signaling pathways. Specifically, we will feature how H_2_O_2_, as a secondary messenger, can induce cysteine-centered oxidative post-translational modifications (oxPTMs); these protein oxPTMs drive downstream signaling pathways and affect cellular processes. In addition, a summary of known cellular signaling pathways that are targeted by mtH_2_O_2_ will be discussed, in the context of neurodegenerative diseases.

## Intracellular sources of hydrogen peroxide production and their regulation

The intracellular balance of ROS is paramount for cell survival and is maintained by reducing excess cellular ROS *via* antioxidants^11^. There are two types of ROS sources within the cell: first, ROS are produced from intracellular biological processes such as OXPHOS and protein disulfide bridge formation^12^. Second, ROS can be produced by external cues such as xenobiotics, microbial invasion, and immune system-derived cytokines^13^; here, ROS production acts to break down foreign entities or induce a downstream signaling pathway. Hence, despite their near-constant production, ROS must be maintained at safe levels.

**Fig. 1:**
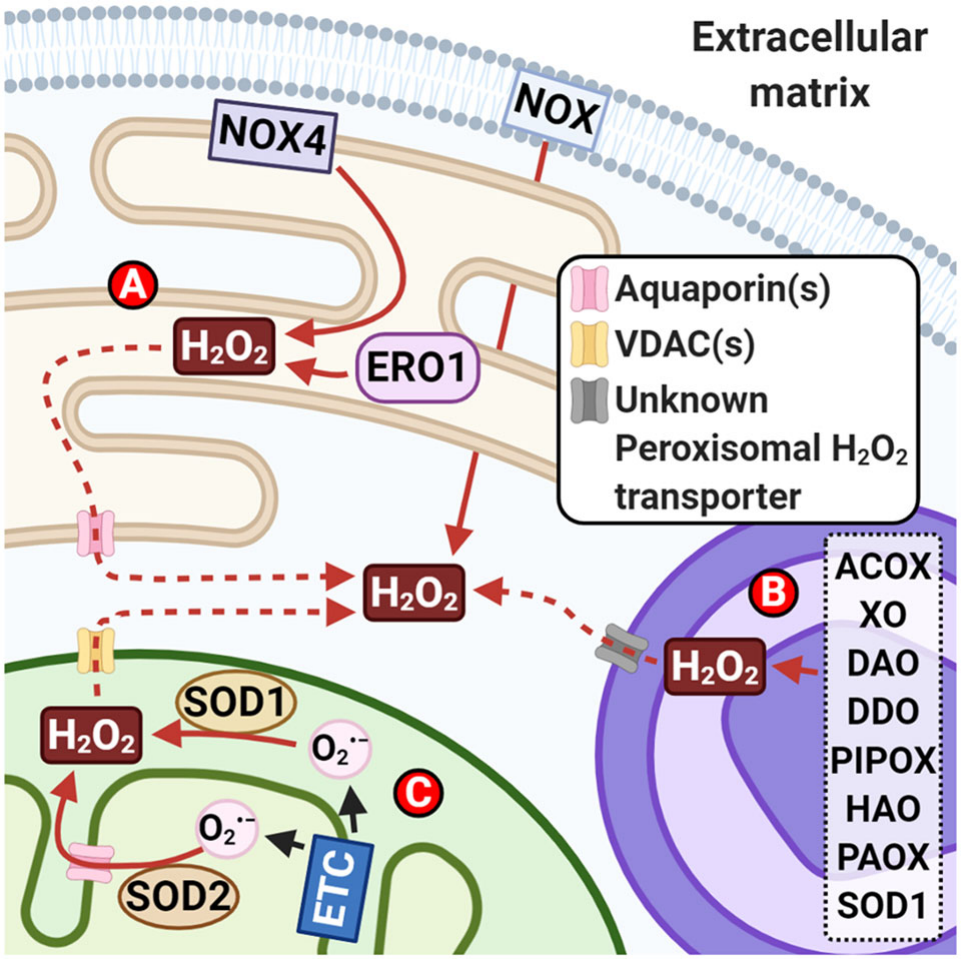
Major sources of cellular reactive oxygen species (ROS) production. The three main producers of cellular ROS are the mitochondria, the endoplasmic reticulum (ER) and the peroxisomes. **A** In the ER, the major source of ER-ROS production is from the process of oxidative protein folding for disulfide bond formation. This process is governed by the enzyme ER oxidoreductin 1 (ERO1). NADPH oxidase 4 (NOX4) is also involved in the production of ER-ROS, mainly ER-H_2_O_2_. **B** In the peroxisomes, the major producer of peroxisomal ROS is the process of beta-oxidation of fatty acids. The main player involved is the enzyme, Acyl-CoA oxidase (ACOX). Numerous other enzymes in the peroxisomes also contribute to the production of peroxisomal ROS: xanthine oxidase (XO); d-amino-acid oxidase (DAO); d-aspartate oxidase (DDO); l-pipecolic acid oxidase (PIPOX); l-α-hydroxyacid oxidase (HAO); polyamine oxidase (PAOX). **C** The main producers of mitochondrial ROS in the mitochondria are from the activity of the electron transport chain (ETC) during oxidative phosphorylation (OXPHOS). The mitochondrial superoxide (O_2_^•−^) produced by the ETC is immediately converted to mitochondrial hydrogen peroxide (H_2_O_2_) by superoxide dismutase 1 and 2 (SOD1 and SOD2). In general, cellular H_2_O_2_ is one of the major and common forms of cellular ROS. Its role as a secondary messenger and its production in these three organelles has raised a suggestion of a potential redox signaling complex among them, highlighting the importance of cellular H_2_O_2_ in cellular redox communications. Created with BioRender.com.

The three main producers of ROS are mitochondria, along with the endoplasmic reticulum (ER), and peroxisome (Fig. 1). In the mitochondrial electron transport chain (ETC), mtROS are produced from the one-electron transfer from O_2_ to the respective electron donors and acceptors, thus generating mtO_2_^•− ^^14^. Owing to mtO_2_^•−^ instability, the presence of nearby antioxidant enzymes—superoxide dismutase 1 (SOD1) and 2 (SOD2)—convert mtO_2_^•−^ to the more stable mtH_2_O_2_^15^. mtH_2_O_2_ can be further reduced to H_2_O by other mitochondrial antioxidant enzymes, such as catalase, glutathione peroxidases (GPXs; isoform 1 and 4), and peroxiredoxins (PRXs; isoform 3 and 5)^16^. Alternatively, mtH_2_O_2_ may exit the mitochondria *via* inner mitochondrial membrane (IMM) channels, such as aquaporins, and diffuse through the porous outer mitochondrial membrane (OMM) into the cytoplasm for potential redox signaling^17–19^.

ER-ROS are produced mainly during protein synthesis^12^ and contribute the greatest to cellular ROS levels^20^. ER-ROS, predominantly as ER-H_2_O_2_, is produced by ER oxidoreductin 1 (ERO1, with α and β subunits) during the disulfide bond formation in oxidative protein folding^21^. In addition, ER-H_2_O_2_ is produced by NADPH oxidase 4 (NOX4), member of the NOX family of membrane-bound NADPH oxidases. NOX4 is the only NOX isoform to directly produce H_2_O_2_, and it has been documented to have many functions, ranging from mediating O_2_ sensing to cell proliferation and differentiation^22,23^. Besides the ER, NOX4 localizes to the nucleus, plasma membrane, and mitochondria^24–26^, though its multiple cellular localization is a source of debate^27,28^. In addition, a detailed review by Chen et al.^27^ describes the multiple roles of NOX4 and highlights H_2_O_2_ as an important secondary messenger. Excessive ER-H_2_O_2_ in the ER is prevented by the activities of various antioxidant enzymes, such as GPXs (isoform 7 and 8) and PRX4^29^.

Peroxisomal ROS, mainly H_2_O_2_, is produced by numerous oxidative enzymes (Fig. 1). For example, a review by Antonenkov et al.^30^ presents fatty acid beta-oxidation by acyl-CoA oxidases as one of the major sources of peroxisomal H_2_O_2_. Akin to the mitochondria and ER, peroxisomes contain antioxidant enzymes such as catalase, SOD1 and PRX5, to maintain the balance of cellular oxidative levels^30^.

The presence of multiple cellular H_2_O_2_ sources and antioxidant enzymes demonstrates that cellular H_2_O_2_ functions beyond toxicity; it is an important secondary messenger that modulates and drives vital downstream signaling pathways, as emphasized in a review by Yoboue et al.^31^. The next section of this review will underscore how cellular H_2_O_2_ can influence signaling pathways by modifying the reactive thiol side chains of the amino acid cysteine.

## Hydrogen peroxide-driven oxPTMs

The complexity of the cellular proteome arises from the presence of post-translational modifications (PTMs), of which numerous types exist, including the well-studied phosphorylation^32^. Commonly, these PTMs modulate protein activity by inducing conformational changes. They may stabilize the protein to avoid degradation or induce the opposite effect. Growing evidence has shown cellular H_2_O_2_ to be a key secondary messenger that can elicit oxPTMs and affect biological processes like cell proliferation^33^.

**Fig. 2:**
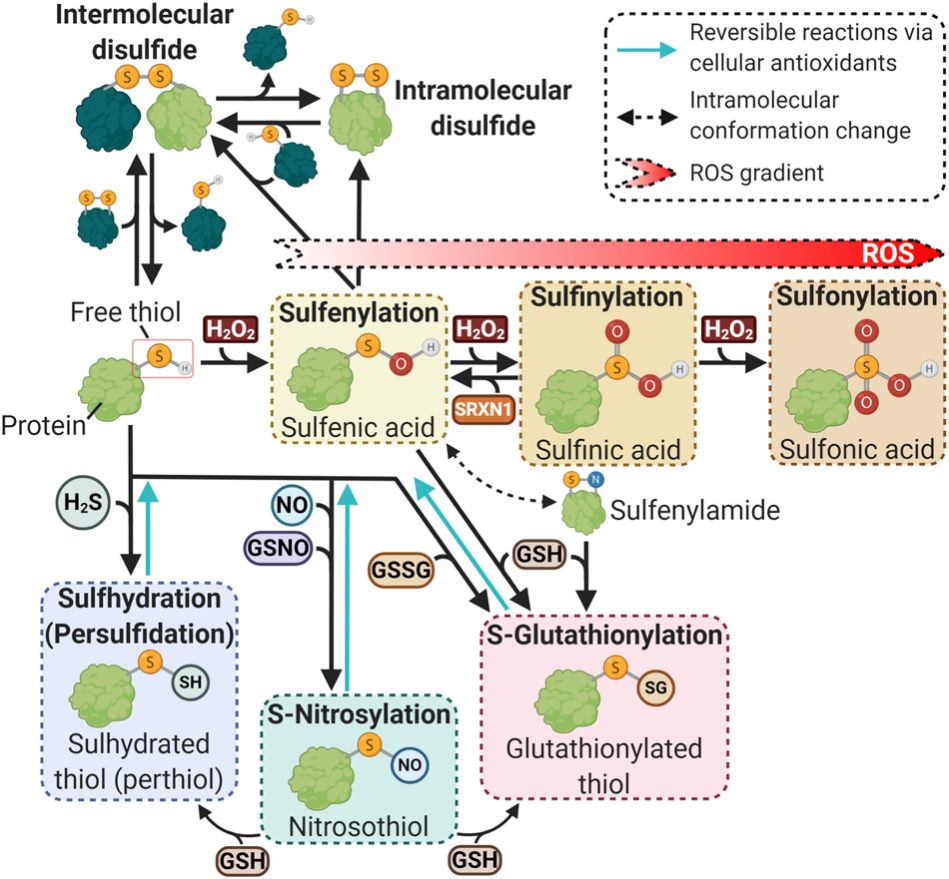
Different types of oxidative post-translational modifications (oxPTMs) to the thiol group on cysteine amino acid in proteins. In the presence of cellular hydrogen peroxide (H_2_O_2_), the free thiol will undergo sulfenylation to form sulfenic acid, which in recent times has been considered an important intermediate in redox signaling. In the presence of increasing oxidative stress, the elevated levels of cellular H_2_O_2_ would lead to the sulfenic acid undergoing sulfinylation to form irreversible sulfinic acid. However, the presence of SRXN1 could convert the sulfinic acid back to sulfenic acid. With even higher concentrations of cellular H_2_O_2_, sulfinic acid can undergo further reaction *via* sulfonylation to form sulfonic acid, which is the final irreversible form of thiol. This highlights the detrimental effects of increasing oxidative stress in cells, which could lead to formation of the irreversible sulfonic acid and the loss of function of important proteins in the cell. Another important thiol oxPTM is the formation of disulfide bonds, both intermolecularly and intramolecularly. The formation of the disulfide bonds is known to potentially activate as well as inhibit the function of the target proteins. Another notable oxPTM is the S-glutathionylation of proteins in the presence of glutathione molecules. Other modifications of free thiol include the sulfhydration by H_2_S, which is an important modification in the central nervous system, and S-nitrosylation that is induced by reaction nitrogen species in the cell. Created with BioRender.com.

The reactive state of ROS can induce chemical modification, altering protein conformation and activity, as observed in other PTMs^34^. Moreover, like phosphorylation, oxPTMs are reversible, demonstrating the potential influence that H_2_O_2_ has on the cellular signaling network^35^. Cellular H_2_O_2_ targets mainly cysteine residues due to their thiol side chains that can be oxidized^36^. Cellular H_2_O_2_ and ROS, along with cellular reactive molecules such as reactive nitrogen species and nitric oxide, can cause the cysteine amino acid to adopt different oxidative states, including S-OH, S-O_2_H, S-O_3_H, and S-NO^37^. Fig. 2 summarizes the complete types of cysteine oxidative state, and the processes that lead to their formation. Such diversity highlights the variation of redox signaling that exists in the cell.

Cysteine oxPTMs include disulfide bonds, where both intermolecular and intramolecular bond formations can occur. Disulfide bonds can occur between thiyl radicals (RS^•^) of two closely placed independent free thiols on the same or separate protein molecule^38^. Alternatively, ROS may convert S-OH groups to RS^•^, thereafter forming disulfide bonds with other thiolates^39^. Disulfide bond formation could lead to changes to protein conformation and function^40^; for instance, the α-subunit of ATP synthase can form a disulfide bridge with the γ-subunit between Cys294 and Cys103 to inhibit ATP production^41^.

Another form of oxPTM is sulfenylation (S-OH), which was previously classified as a toxic byproduct from cellular H_2_O_2_ and O_2_^•−^ reactions; however, researchers in the last decade have identified it as an important intermediate in redox signaling pathways^39^. The reactive and unstable nature of S-OH enables its conversion into other types of oxPTMs in the presence of reduced glutathione (GSH). S-glutathionylation, as reviewed by Zhang et al.^42^, is a reversible process that depends on the presence of cellular antioxidants; it thus protects the target protein by preventing irreversible oxPTMs^39^. In addition, S-OH can react with free thiols of a target protein to form disulfide bonds. Such plasticity in protein modification demonstrates how oxPTMs can help drive redox signaling.

In extremely high levels of H_2_O_2_, oxPTMs can become an irreversible process, as the oxidative state of cysteine shifts beyond sulfenylation to sulfinylation and eventually, to sulfonylation (Fig. 2). Although sulfonylation is classified as irreversible, it is possible to reverse sulfinylation *via* sulfiredoxin-1; this process forms the reversible sulfenic acid form of cysteine^43,44^. The progression of cysteine to its irreversible form illustrates the importance of cellular ROS levels: Low levels of cellular ROS would initiate oxPTMs, whereas excessive levels of cellular ROS would irreversibly oxidize proteins and cause deleterious consequences. A review by Chung et al.^45^ delineates the capability of cysteine-based oxPTMs in redox signaling and physiological processes, particularly in the highly oxidized cardiovascular environment.

The ability of cellular H_2_O_2_ to induce oxPTMs is a key component in redox signaling^33^, especially when cysteine residues form only 2% of the cellular proteome but is involved in the most PTMs^46^. Hence, understanding how H_2_O_2_ influences the biological signaling pathways in various diseases facilitates potential therapeutic interventions, particularly in neurodegenerative diseases, due to their close association to increased oxidative stress. In the next section of this review, we will highlight biological mechanisms that can be influenced by cellular H_2_O_2_, especially those associated with the mitochondria.

## Mitochondria-associated biological processes regulated by potential mitochondrial hydrogen peroxide-mediated redox signaling

Besides producing ATP during OXPHOS, mitochondria also play an important role in triggering and regulating apoptosis, as documented by Orrenius et al.^47^. Lower levels of ROS were shown to activate cellular survival responses, while higher levels of ROS activate death processes^48^. In the intrinsic apoptotic pathway, mitochondrial outer membrane permeabilization—a process regulated by the B-cell lymphoma-2 (BCL-2) protein family, such as the Bcl-2-associated X protein (BAX)—releases numerous proapoptotic proteins from the mitochondrial intermembrane space, including cytochrome c (Cyt c)^49^. Cyt c can trigger rapid oligomerization of apoptotic protease-activating factor-1, which recruits caspase-9 into the apoptosome to cleave and activate downstream effectors like caspase-3^50^. Moreover, Cyt c release triggers the production of mtROS, suggesting a role of mtROS in redox signaling^51^. H_2_O_2_-mediated oxPTM of BAX can induce protein disulfide dimerization and translocation to the OMM^52^; this was seen in human colon adenocarcinoma cells upon Cys62 modification of BAX by H_2_O_2_^53^. In addition, apoptotic caspase activity can be redox-regulated by oxPTM, with procaspase-9, procaspase-3, and caspase-3 susceptible to S-glutathiolation^54^. As detailed by Benhar^55^, ROS such as H_2_O_2_ can contribute to oxPTM of proteins involved in the apoptotic pathway. However, whether these events are regulated by mtH_2_O_2_ remain unknown. Neurodegenerative diseases, such as Alzheimer’s disease (AD), are associated with the accumulation of misfolded proteins, like β-amyloids, that can cause neuronal death via oxidative stress^56^. However, it remains debatable whether ROS mainly causes cell death or could contribute to redox signaling of important survival pathways.

**Fig. 3:**
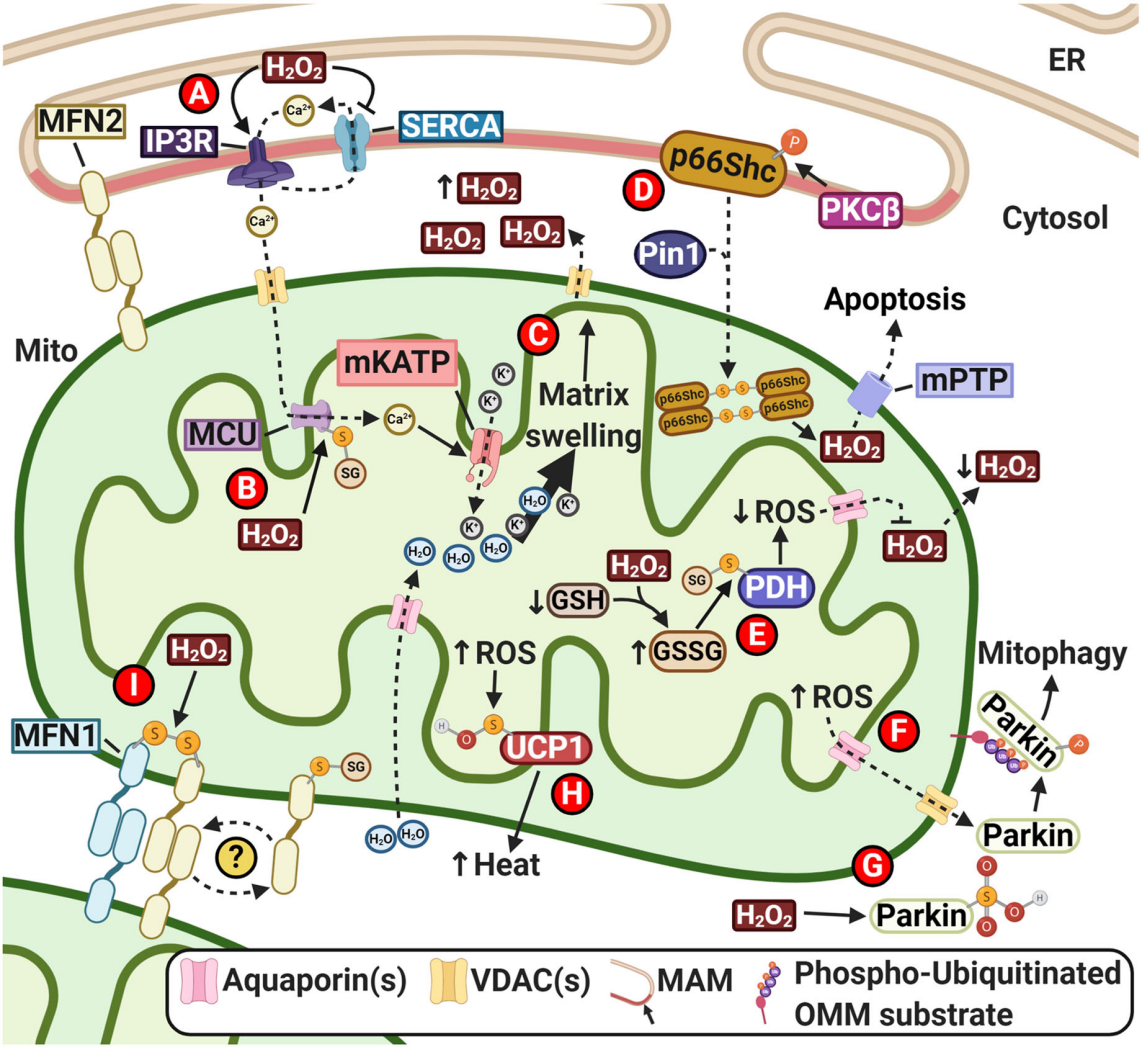
Known redox signaling pathways regulated by mitochondrial hydrogen peroxide (mtH_2_O_2_). **A** Calcium (Ca^2+^) signaling communicated between the endoplasmic reticulum (ER) and the mitochondria (Mito) *via* the inositol 1,4,5-trisphosphate receptor (IP3R), sarco-/endoplasmic reticulum Ca^2+^-ATPase (SERCA) Ca^2+^ channels, and the voltage-dependent anion channel (VDAC); MAM: Mitochondria-associated membrane. **B** Ca^2+^ uptake by mitochondrial calcium uniport (MCU) after S-glutathionylation of Cys97. **C** mtH_2_O_2_ efflux from mitochondrial matrix swelling and reduced intermembrane space. **D** Cell death induced by the opening of the mitochondrial permeability transition pore (mPTP) *via* the tetramerization of p66Shc; PKCβ: Protein Kinase C β; Pin1: Prolyl Isomerase 1. **E** Increasing oxidized glutathione (GSSG) to reduced glutathione (GSH) ratio could reduce mtROS production by pyruvate dehydrogenase (PDH) and reduce the release of mtH_2_O_2_. **F** Parkin-dependent mitophagy mediated by mtROS; OMM: Outer mitochondrial membrane. **G** Increased levels of cellular ROS have been known to cause Parkin sulfinylation and inhibition by intracellular H_2_O_2_. **H** Increased heat production by sulfenylated uncoupler protein 1 (UCP1) during hypothermia. **I** Activation of mitofusion from the disulfide bond formation of mitofusin 1 (MFN1) and mitofusin 2 (MFN2). This disulfide bond formation could further S-glutathionate MNF2 to activate mitofusion. Created with BioRender.com.

The mitochondrial enzyme pyruvate dehydrogenase (PDH) catalyzes pyruvate to enable its entry into the Krebs cycle; however, PDH is also a major contributor of mtH_2_O_2_ production in various tissues, approximately four times more than Complex I of the ETC^57^, thus suggesting a role of PDH in redox signal modulation. A recent study done by O’Brien et al.^58^ demonstrated that as mtH_2_O_2_ increases, so does oxidation of GSH to glutathione (GSSG). The increase of GSSG leads to PDH S-glutathionylation, causing a reduction in mtH_2_O_2_/O_2_^•−^ production (Fig. 3E). This observation suggests that PDH can control the changing mitochondrial redox status and regulate the emission of mtH_2_O_2_. Interestingly, when reverse electron transfer occurs in the ETC under stress conditions, S-glutathionylation led to an increased production of mtH_2_O_2_/O_2_^•−^ by PDH, potentially leading to mitochondrial dysfunction and cellular oxidative stress. This bi-directional effect of PDH S-glutathionylation on mtROS production may have implications in oxidative stressed-related diseases such as neurodegenerative diseases.

Another study of mtH_2_O_2_-mediated biological process occurred in brown adipose tissue (BAT) by Chouchani et al.^59^. The authors found that cold-induced BAT thermogenesis was triggered by an increase in mtROS. Conversely, mtROS diminution in BAT using antioxidants mitoQ and N-acetylcysteine (NAC) led to hypothermia during cold exposure. The authors identified Cys253 sulfenylation on uncoupling protein 1 (UCP1), which is part of the mitochondrial respiratory system (Fig. 3H); this oxPTM sensitizes UCP1 to adrenergic activation in BAT, increasing UCP1-mediated thermogenic respiration and ATP expenditure, which then stimulates increased heat production. Contrarily, the scavenging of mtROS during cold exposure abrogated UCP1-mediated thermogenesis. This study affirms the significance of mtROS level in redox signaling. Although the authors did not mention mtH_2_O_2_ in the oxPTM of UCP1, the accepted role of H_2_O_2_ in sulfenylation implies its involvement here^60^. These examples highlight the influence of mtH_2_O_2_ as a secondary messenger in cellular pathways.

The development of neurodegenerative diseases may stem from mitochondrial loss of function mediated by excessive production of ROS; however, antioxidant treatment to combat neurodegenerative diseases received disappointing results, potentially due to the loss of these mtH_2_O_2_-driven biological processes required for cellular recovery^61^. Moreover, studies have also shown that increased mtROS did not negatively impact longevity, suggesting an important role of mtROS in age-related diseases such as neurodegenerative diseases^62^.

Cellular survival necessitates mitochondrial quality control (MQC); mitochondrial dysfunction can lead to cell death upon the accumulation of many apoptotic mediators in the mitochondria, as suggested in AD^63^. Hence, to safeguard mitochondrial integrity, mitochondrial dynamics (fission and fusion) exist, and their dysregulation could lead to neurodegenerative diseases^64^. Through mitophagy, dysfunctional mitochondria can be removed from the cell. Interestingly, there is evidence showing the involvement of mtROS in the process of mitochondrial fusion (mitofusion). A study conducted by Thaher et al.^65^ identified mitofusin 2 (MFN2) to undergo oxPTM and form disulfide bonds with adjacent MFN1 to induce mitofusion (Fig. 3I). The authors found that the Cys684 residue in MFN2 is key in this redox-regulated mitofusion and suggested that S-glutathionylation of Cys684 could also play a role in MFN2-mediated MQC in response to the changes in the intracellular redox environment. Accordingly, Mailloux and Treberg^66^ proposed the existence of a mitochondrial metabolism-linked redox signal that is indirectly mediated by mtH_2_O_2_ *via* the glutathione redox buffering system. Moreover, our lab recently demonstrated that mtROS could induce Parkin/PINK1-dependent mitophagy during mitochondrial stress^67^. We demonstrated that the increased mtROS from VDAC1 overexpression led to Parkin recruitment to the mitochondria (Fig. 3F), and this effect was abrogated by NAC and catalase. Although we did not further probe for oxPTMs, our results suggest that Parkin/PINK1-mediated mitophagy could be redox-sensitive, and there could be some form of redox signaling involved in the mitochondrial turnover mechanism. Interestingly, the cysteine-rich regions of Parkin can be sulfonylated by cellular H_2_O_2_, leading to the loss of its E3 ligase activity (Fig. 3G), which may be a contributing factor in Lewy bodies formation in Parkinson’s disease (PD)^68^. As such, these studies show that mitochondrial redox signaling might play a role in MQC, whose dysfunction has been implicated in the pathogenesis of PD.

## Redox communication between the ER and the mitochondria

Apart from the redox signaling system, mitochondria also mediate calcium (Ca^2+^) signaling, which is involved in many diverse physiological processes such as muscle contraction and neuronal excitation^69^. Ca^2+^ imbalance can trigger cell death *via* apoptosis^70^. The exchange of Ca^2+^ between the ER and mitochondria occurs at the mitochondria-associated membrane (MAM) (Fig. 3A)^71^. A study by Booth et al.^72^ showed that the presence of ER-H_2_O_2_ at the MAM can lead to the inactivation of sarco-/ER Ca^2+^-ATPase (SERCA) and activation of inositol 1,4,5-trisphosphate receptor (IP3R). These events induce an influx of Ca^2+^ into the mitochondria, thereafter causing an influx of K^+^ and H_2_O into the mitochondrial matrix (MM). Consequently, MM swelling occurs (Fig. 3C), reducing the volume of the mitochondrial intermembrane space (IMS) and releasing mtH_2_O_2_ into the interface region between the MAM and the mitochondria. mtH_2_O_2_-containing redox nanodomains are formed; however, their function is not known, according to the authors. Additionally, mitochondrial Ca^2+^ uniporter (MCU) can be S-glutathionylated at Cys97 by mtH_2_O_2_ to increase Ca^2+^ uptake (Fig. 3B). Thus, these examples highlight the potential redox signal exchange between the ER-MAM and the mitochondria.

Despite being largely localized in the cytoplasm, recent studies have identified p66Shc to be associated to the MAM^73^. Oxidative stress induces p66Shc phosphorylation by protein kinase C β (PKCβ), triggering p66Shc interaction with prolyl isomerase 1 (Pin1) and subsequent mitochondrial translocation (Fig. 3D)^74^. A study by Gertz et al.^75^ highlighted the presence of a dimer-tetramer shift of p66Shc, where tetrameric p66Shc could trigger apoptosis by generating mtH_2_O_2_ and opening the mitochondrial permeability transition pore (mPTP). Gertz and Steegborn^76^ suggested that tetramerization increases p66Shc affinity to mPTP, and the local production of mtH_2_O_2_ opens mPTP to trigger apoptosis. They also remarked that tetrameric p66Shc could, *via* an unknown process, increase mtH_2_O_2_ levels enough to trigger mPTP opening and apoptosis. Therefore, a possible question is whether a mechanism to control the amount of mtH_2_O_2_ produced by the p66Shc tetramer exists. Indeed, Gertz et al.^75^ demonstrated that thioredoxin and glutathione can abrogate the apoptosis triggered by tetrameric p66Shc. These results highlight the important interplay between mitochondrial antioxidant system and the level of mtROS, simultaneously raising the question on how cells determine the level of cellular ROS required for redox signaling transduction.

The above redox signaling between the ER-MAM and mitochondria illustrates the existence of inter-organelle redox signaling regulation. Notably, there are growing studies to suggest MAM might play a role in the pathogenesis of neurodegenerative diseases, as reviewed by Rodríguez-Arribas et al.^77^. Considering the potential existence of the redox nanodomains, MAM’s association in neurodegenerative and age-related diseases might be of interest to determine whether this pocket of mitochondrial-derived redox signaling might influence the pathogenesis of neurodegenerative diseases.

## Mitochondrial hydrogen peroxide and their potential role in the pathogenesis of neurodegenerative diseases

The brain consumes nearly a quarter of the body’s total intake of glucose and O_2_^78,79^, with 20% of the total O_2_ uptake driving ATP-producing OXPHOS. The brain’s high metabolic level produces high levels of ROS, making brain tissue the most vulnerable tissue to oxidative damage^80^. Indeed, the mitochondrial and free-radical theories of aging attribute the brain’s high cellular ROS levels to oxidative damage and aging^80,81^; however, recent and growing evidence shows cellular ROS as essential signaling molecules, especially with cellular H_2_O_2_-associated oxPTMs.

This paradigm shift is derived mainly from model-based organism studies with invertebrates such as *Caenorhabditis elegans* and yeast. For example, a study by Schulz et al.^82^ demonstrated that glucose deprivation increases OXPHOS and mtROS, and delays *C. elegans* aging. Similarly, a recent study by Wang et al.^83^ showed that lifespan extension conferred by the increased mtROS was diminished by glucose *via* reducing cellular ROS levels. Other studies echo this concept by identifying the increase in mtROS as a common aftermath of numerous conserved longevity-promoting interventions, thus originating the term “mitohormesis”^84,85^. Mitohormesis is the process of mitochondrial adaption from increased mtROS exposure, activating stress resistance mechanisms to improve anti-aging effects^85^. As the brain consumes a high volume of glucose for energy production, the detrimental effect of glucose on aging and neurodegenerative diseases could stem from mitochondrial dysfunction, as highlighted by Cheng et al.^86^. The disruption of redox balance and glucose regulation in dysfunctional mitochondria could lead to the development of neurodegenerative diseases. In addition, a recent study by Smith et al.^87^ demonstrated that the GSH redox pathway can control mitochondrial shape in axons of *Drosophila*. Collectively, these observations accentuate the importance of “healthy” mitochondria in preventing age-related diseases like neurodegenerative diseases.

ROS formation (mainly O_2_^•−^) increases with increasing O_2_ concentration^88–91^. However, studies have revealed that ROS levels are either constant or elevated with declining O_2_ levels^92,93^. Moreover, aging and neurodegenerative diseases are closely linked to hypoxia^94^. Despite the hypoxic decline of O_2_ levels in aging, O_2_ to the brain is not completely depleted, but the state of chronic hypoxia increases oxidative stress and trigger apoptosis and neurodegeneration^94^. In a review, Waypa et al.^95^ explored the possibility that mtROS are O_2_ sensors during hypoxia. Previous studies have shown intracellular H_2_O_2_ levels reversibly increase during hypoxia^96–99^; inhibiting mitochondrial complex III-mediated ROS production during hypoxia impeded the stabilization of hypoxia inducible factor-1 subunit alpha (HIF-1α) and affected the cellular response to hypoxia^100,101^. These observations emphasize the importance of functional mitochondria, failure of which could explain the manifestation of neurodegenerative diseases.

A review by Shadel and Horvath^102^ illustrated many important mtROS signaling pathways in reversing aging processes: particularly, the relationship between UCP2 and mtROS in controlling energy metabolism of the brain. During starvation, the increase in mtROS production from lipid oxidation can promote UCP2 activity to preserve low ROS levels while maintaining energy production^103^. In the well-fed state, glucose OXPHOS increases mtROS production^104^, which drives leptin and insulin to exert the systemic equivalent of satiety^103,105–108^. Importantly, disruption to such mitochondrial redox signaling would impair these physiological processes;^103,106,108,109^ this highlights the importance of mtROS and may explain the near-zero success rates in antioxidant therapies for neurodegenerative diseases^61^. Effective neurodegenerative disease treatment thus lies in redox homeostasis rather than the absolute amount of cellular ROS. The solution should restore redox balance and redox signaling, maintaining the correct ROS type, location in the signaling pathway, and amounts.

A hallmark of aging is the accumulation of damaged mitochondria in the brain; particularly, ETC activity is disrupted as aging progresses^110–115^. Decreased Complex IV activity increases mtROS production, whereas reduced Complex I activity affects cellular differentiation. Interestingly, mtROS produced by Complex I enters the MM, whereas mtROS by Complex III enters the IMS and is involved in site-specific redox signaling (such as HIF-1α signaling, as previously highlighted)^116^. These differences imply site-specific mitochondrial redox signaling pathways exist, which are disrupted by the accumulation of damaged mitochondria and subsequent elevation of mtROS levels.

Two types of ROS exist: low-reactive ROS (such as O_2_^•−^ and H_2_O_2_), and high-reactive ROS (such as hydroxyl radicals and peroxynitrites, produced by H_2_O_2_ undergoing Fenton’s Haber–Weiss reactions)^117,118^. Although low-reactive ROS act as secondary messengers, high-reactive ROS contribute to oxidative damage and cell death. A study by Forster et al.^119^ showed oxidative stress to correlate with loss of cognitive ability in rodents, whereas neuronal cell death initiates brain aging and triggers neurodegenerative diseases^120^. However, mtROS should not be synonymized with oxidative stress and neuronal death, as we continuously emphasize the role of mtROS in redox signaling.

The aforementioned discussions, along with a review by Angelova and Abramov^121^, point out mitochondrial dysfunction as a pathological cue to the progression of various neurodegenerative diseases. Although damaged mitochondria are usually removed by quality control processes such as mitophagy and proteasomal degradation^122^, aging could reduce the efficiency and quality of these corrective processes, causing gradual accumulation of “unhealthy” mitochondria. Studies have shown that increasing the efficiency of proteasomal activities and mitophagy could increase longevity of various model organisms including worms, flies, and mice^123–126^. Moreover, Chen et al.^127^ highlights the importance of mitophagy in the pathology of neurodegenerative diseases. Although there is no concrete evidence yet to suggest that maintaining a pool of healthy mitochondria is key to reducing the negative aspects of aging, the manifestation of neurodegenerative diseases through such a scenario might still hold true. A review by Stefanatos and Sanz^110^ suggests that specific mtROS, like mtH_2_O_2_, are produced in specific locations and amounts to drive site-specific redox signaling in healthy mitochondria; conversely, “unhealthy” mitochondria produce ROS of the wrong type, location, or amount, which could derail redox signaling. This is highlighted in our previous study showing mitophagy activation by mtROS, suggesting that understanding the complexity of redox signaling in the brain, and how they are alternated in neurodegenerative diseases are beginning to be of importance to further our understanding of their pathogenesis and identify correct therapeutic interventions.

The studies highlighted thus far demonstrate the importance of mtROS in redox signaling, though the type of ROS is rarely specified. A review by Mailloux^128^ explains that mtO_2_^•−^ must be removed swiftly to prevent the deactivation of Fe-S cluster-containing proteins in the mitochondria; the rapid conversion of mtO_2_^•−^ to mtH_2_O_2_ maintains its concentration at ρM range^14^. Therefore, the dominant mtROS in the matrix is mtH_2_O_2_. Apart from mtO_2_^•−^, mitochondria also have an extensive system of removing mtH_2_O_2_; in addition, this system acts as a sink for cellular H_2_O_2_, as suggested by Mailloux. A study by Dey et al.^129^ highlights the importance of the mitochondrial antioxidant defenses, wherein the loss of the mitochondrial antioxidant defenses led to the dysregulation of the cytosolic H_2_O_2_ redox environment. This study emphasizes the role of mitochondria in governing the cellular levels of H_2_O_2_, thus demonstrating the mitochondria’s important role in modulating cellular H_2_O_2_ signals. Therefore, it is important to further our understanding in the events how dysregulation of mtH_2_O_2_ and cytosolic H_2_O_2_ in neurodegenerative diseases could lead to their pathologies.

## Conclusion and future perspectives of mitochondrial and ER-derived hydrogen peroxide in neurodegenerative diseases

Although current therapeutic approaches against neurodegenerative diseases lack success, continuous research has enhanced understanding of such diseases. Mitochondrial dysfunction has been identified as an important hallmark, and ROS as important secondary messengers. Furthermore, a key aspect of neurodegenerative diseases pathogenesis has been suggested: the potential and paramount role of redox signaling.

**Fig. 4:**
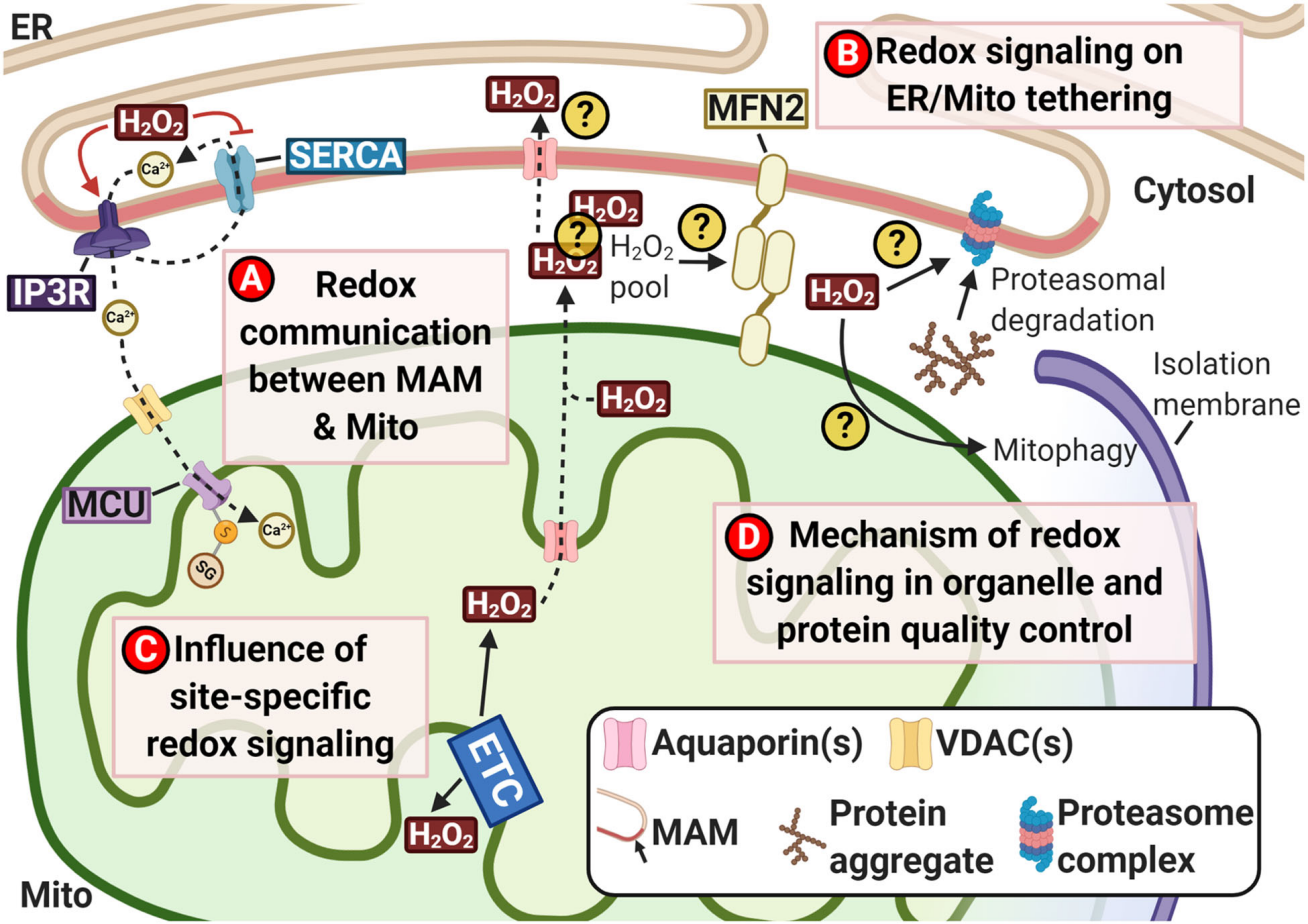
The overview schematics and future directions of the importance of redox communication in the cell leading to pathogenesis of neurodegenerative diseases. **A** Investigate how the dysregulation of redox-regulated calcium (Ca^2+^) signaling between the endoplasmic reticulum (ER) and the mitochondria (Mito) could contribute to the pathogenesis of neurodegenerative diseases; MAM: mitochondria-associated membrane. **B** Understand how the release of redox signals such as mitochondrial hydrogen peroxide (H_2_O_2_) into the MAM-mitochondrial interface could affect ER-mitochondria tethering and dysregulate mitochondrial dynamics that could contribute to the pathogenesis of neurodegenerative diseases. **C** Understand how site-specific redox signaling in the mitochondria could be mediated by the levels of intracellular ROS and determine the presence of H_2_O_2_ pool that can act as a reservoir of secondary messengers to trigger different signaling pathways throughout the whole cells. **D** Identify the underlying redox regulation of mitochondrial quality control and its significance to the pathogenesis of neurodegenerative diseases. Created with BioRender.com.

We mentioned earlier that the presence of communication between the MAM at the ER and mitochondrial OMM interface was identified in recent studies, and that H_2_O_2_ could affect the Ca^2+^ exchange between ER and mitochondria (Fig. 4A). Ca^2+^ signaling has been implicated in various neurodegenerative diseases, where their protein aggregates can target various Ca^2+^ channels and affect their Ca^2+^ flux^130^. For instance, α-synuclein (α-syn), the major aggregated protein in PD pathology, reportedly associates with MAM^131^. However, the pathological implication of this association has not been found. As Ca^2+^ is known to play a role in the ER during protein folding^132^, and aberrant protein aggregation characterizes AD and PD, disrupted Ca^2+^ signaling may promote disease-associated protein misfolding and aggregation. Furthermore, previous studies showed Ca^2+^ to partake in cellular quality control such as autophagy^133^, further tying the dysregulation of Ca^2+^ signaling to the accumulation of protein aggregates. Therefore, understanding the relationship of Ca^2+^ and redox signaling could help better understand their roles in neurodegenerative diseases.

Besides Ca^2+^ and redox signaling, another area of interest is the potential role of mitochondria tethering to the MAM, which can modulate mitochondrial morphology. As highlighted previously, oxPTMs of MFN2 by H_2_O_2_ can initiate mitofusion (Fig. 4B). Notably, we highlighted that mtH_2_O_2_ release to the MAM-mitochondrial interface during MM swelling (Fig. 3C) could generate a pool of H_2_O_2_ as secondary messengers for redox signaling. If this pool exists, it could affect various signaling pathways like Ca^2+^ signaling. Furthermore, this pool of mtH_2_O_2_ could affect mitochondria tethering to the MAM, thus affecting mitochondrial morphology and recycling, including the clearance of “unhealthy” mitochondria and generation of “incorrect” ROS types. Therefore, whether α-syn association with MAM disrupts mitochondrial turnover and homeostasis in PD could be of interest to study.

As suggested previously, the existence of site-specific redox signaling by mtROS could trigger different processes. Some of these known site-specific ROS-driven processes (like cell growth and differentiation) are involved in nucleus signaling, suggesting mtROS secretion to the MAM-mitochondrial interface to create a ROS pool, as mentioned above (Fig. 4C). Transmission of the redox signal could occur: first, from the mitochondria to the ER then to the nucleus; secondly, mitochondria tethering to an ER region near the nucleus; or thirdly, mitochondria tethering directly to the nucleus. Interestingly, a recent study by Pak et al.^134^ debunked the existence of H_2_O_2_ efflux from the mitochondria to the cytosol despite identifying the existence of intracellular H_2_O_2_ gradient; however, the experiments were done using K562 lymphoblast cells instead of a normal cell line. Therefore, replicating the experiments in cell lines such as neurons will give insight into the existence of a mtH_2_O_2_ pool in the MAM-mitochondrial interface and its role in neurodegenerative diseases.

The “inaccurate” redox signal could also affect cellular quality controls, such as proteasomal degradation of misfolded or aggregated proteins, or organellar quality control, such as mitophagy (Fig. 4D). In the latter case, our previous study showed that ROS could trigger mitophagy directly, demonstrating that mitophagy could also be a redox-sensitive process. Further research on the regulation of cellular quality control by redox signaling would aid the identification of diagnostic and therapeutic targets in neurodegenerative diseases.

Evidently, the involvement of ROS in aging and neurodegenerative diseases must be further investigated to elucidate disease pathogenesis. One approach is to identify proteins that undergo cysteine oxPTMs and map the neuronal redox signaling pathways. Through this, disruptions to redox signaling pathways in neurodegenerative diseases can be identified. Various methods to identify proteins with oxPTMs were reviewed elsewhere^42^; these assays use thiol-labeling chemicals such as iodoacetamide^135^ to identify proteins with thiol groups oxidized by intracellular redox signaling. Coupled with an appropriate reporter such as biotin, the oxPTMed proteins can be identified after pulldown and mass spectrometry (MS). Most of these methods operate under the assumption that the assay will not react to oxidized thiols of target proteins. However, a major disadvantage of these approaches is the lack of quantification. For instance, a 10-fold change in oxPTMs may influence little if it curtails 0.1–1% of the intact protein level, compared with a 10-fold change representing 10–100% of the intact protein level. Therefore, quantitative proteomics has been used to address this issue *via* the use of labels such as isotope-coded affinity tag (ICAT).

ICAT is similar to the above approaches but utilizes isotopic tags to differentially label samples. The mass difference of the isotopic tags is detected and quantified using MS, thus determining the level of oxPTMed cysteine of the target protein. This approach has been used for over a decade, and Topf et al.^136^ demonstrated recently on its use for their study to identify redox switches modulating the global protein translation in yeast triggered by mtROS. However, ICAT detects only the site of oxPTM, not the type. In addition, S-OH is considered a key redox switch in the field of oxPTMs, thus methods to identify such intermediates were developed^137,138^. Most approaches thus far utilize chemical derivatization or trapping to induce reactions with S-OH, forming a stable thioether adduct^139–141^ that can then be quantified using MS or *via* a fluorescent or biotinylated tag^142–145^.

Besides the identification of oxPTMed proteins, identifying the localization of cellular ROS, particularly mitochondrial and ER-H_2_O_2_, can give greater insight into redox signaling. As highlighted in a methods paper by Oparka et al.^146^, the ROS-reactive fluorescent probe 2´,7´-dichlorodihydrofluorecein diacetate (H_2_DCFDA) and its derivatives have been widely used as a H_2_O_2_-specific probe in intact cells. However, H_2_DCFDA is not specific to H_2_O_2_. A H_2_O_2_-specific biosensor, termed HyPer, was subsequently developed from circularly permutated YFP inserted into the H_2_O_2_-sensitive OxyR-regulatory domain from *Escherichia coli*^147–149^. As HyPer is both H_2_O_2_ and pH-sensitive, a parallel control using SypHer is required, as it is only pH-sensitive but possesses the same spectral properties^150–152^. Furthermore, these H_2_O_2_-biosensors are insensitive to smaller changes to H_2_O_2_ concentration near the basal oxidant level^134^. Fortunately, Pak et al.^134^, in a recent study, developed a new HyPer variant, termed HyPer7, using circularly permutated GFP inserted into the OxyR-regulatory domain from *Neisseria meningitidis*. This H_2_O_2_-biosensor is ultrasensitive, ultrafast, and pH-stable. Using this probe, the authors identified the existence of H_2_O_2_ gradients within the cell and evaluated the H_2_O_2_ movement between the mitochondria and cytosol.

Despite the improving technology, much remains lacking for reliable and robust methodology in redox signaling, including the absolute quantification of oxidized proteins that can be mapped onto complex biological systems. Before such a system is in place, we will continue to rely on current methods, preferably utilizing multiple approaches to improve the accuracy of our research data on identifying the redox signaling of neurodegenerative diseases.
